# Reducing delayed transfer of care in older people: A qualitative study of barriers and facilitators to shorter hospital stays

**DOI:** 10.1111/hex.13588

**Published:** 2022-10-03

**Authors:** Helen Smith, Chloe Grindey, Isabel Hague, Louise Newbould, Lesley Brown, Andrew Clegg, Carl Thompson, Rebecca Lawton

**Affiliations:** ^1^ Improvement Science Theme, NIHR Applied Research Collaboration Yorkshire and Humber Bradford Institute for Health Research Bradford UK; ^2^ Social Policy Research Unit (SPRU) University of York York UK; ^3^ Academic Unit for Aging and Stroke Research Bradford Institute for Health Research Bradford UK; ^4^ School of Healthcare, Faculty of Medicine and Health University of Leeds Leeds UK; ^5^ School of Psychology University of Leeds Leeds UK

**Keywords:** discharge to assess, hospital discharge, hospital stay, older people, transfer of care

## Abstract

**Introduction:**

Growing numbers of older patients occupy hospital beds despite being ‘medically fit’ for discharge. These Delayed Transfers of Care amplify inefficiencies in care and can cause harm. Delayed transfer because of family or patient choice is common; yet, research on patient and family perspectives is scarce. To identify barriers to, and facilitators of, shorter hospital stays, we sought to understand older people's and caregivers' thoughts and feelings about the benefits and harms of being in hospital and the decisions made at discharge.

**Methods:**

A multimethod qualitative study was carried out. Content analysis was carried out of older people's experiences of health or care services submitted to the Care Opinion online website, followed by telephone and video interviews with older people and family members of older people experiencing a hospital stay in the previous 12 months.

**Results:**

Online accounts provide insight into *how care was organized* for older people in the hospital, including deficiencies in care organization, the discharge process and communication, as well as *how care was experienced* by older people and family members. Interview‐generated themes included shared meanings of hospitalization and discharge experiences and the context of discharge decisions including failure in communication systems, unwarranted variation and lack of confidence in care and lack of preparation for ongoing care.

**Conclusion:**

Poor quality and availability of information, and poor communication, inhibit effective transfer of care. Communication is fundamental to patient‐centred care and even more important in discharge models characterized by limited assessments and quicker discharge. Interventions at the service level and targeted patient information about what to expect in discharge assessments and after discharge could help to address poor communication and support for improving discharge of older people from hospital.

**Patient or Public Contribution:**

The Frailty Oversight Group, a small group of older people providing oversight of the Community Aging Research 75+ study, provided feedback on the research topic and level of interest, the draft data collection tools and the feasibility of collecting data with older people during the COVID‐19 pandemic. The group also reviewed preliminary findings and provided feedback on our interpretation.

## 1 INTRODUCTION

Patients experiencing Delayed Transfer of Care (DToC)—often termed ‘bedblockers’^1^, ^2^—occupy hospital beds despite being declared ‘medically fit’ for discharge^3^, ^4^ Around 2.3 million excess days are generated by such delays and numbers are increasing: 25% higher in 2017 than 2016.^5^ DToC means fewer elective procedures and NHS Trusts incurring costs for patients who are ready to leave hospital—DToCs cost providers an estimated £173 million in 2016/17.^5^

DToC may have detrimental physical and emotional effects for hospitalized older people. Functional decline and increased frailty occur in a third of older patients.^6^ Patients with functional decline or frailty can become ‘trapped’ in hospital beds, and their more complex needs at the time of discharge place greater reliance on finite social or community support.^1^, ^7^, ^8^

Hospitals can of course improve older patients' health and provide rehabilitation to increase independence and overall quality of life. While hospitalization benefits may tail off with extended stays,^9^ conceptualizing ‘benefit’ as more than ‘clinical effectiveness’ and incorporating patients feeling cared for, safe, helped to manage at home and relief for families suggests that positive effects are possible.^10^ These positive aspects are not trivial; feeling safe can increase hope and control and feeling better can be a function of improved physical condition generally as well as specific symptom resolution.^11^, ^12^ Feelings of greater control also impact on choices.^13^ Research from a patient and family perspective tends to focus on the impact of hospital stays on older people and caregivers; there is an evidence gap specifically on the decisions made around the time of discharge and their relationship with the perceived benefits and harms of extended stays.

Policies catalysed by the COVID‐19 pandemic, such as ‘discharge to assess,’^14^ whereby assessments of care needs take place at home or in the community, have been variably implemented and their effects on outcomes are uncertain. Simply shifting the locus of decision‐making from a clinical setting to a domiciliary one, however, ignores differences between and within system elements and supra‐organizational units such as integrated care systems. Consequently, historic inequalities in processes, care and outcomes for older people could be perpetuated and even exacerbated.^15^

Sixteen percent of DToC in England can be attributed to carer or familial choice, behaviours^4^ or lack of awareness. Examples include preferences for specific care homes, refusing alternatives offered, difficulty attending planning meetings,^16^ being unaware of the cost implications of care, unavailability due to prearranged holidays, refusing to accept equipment and familial conflict around discharge plans.^17^ Age UK (the UK's largest charity for older people) suggests that family‐attributed causes of DToC are more likely to represent reasonable objections, inadequate communication or lack of decisional involvement.^18^

We sought to understand older people's and caregivers' thoughts and feelings about the benefits and harms of being in hospital and the decisions made at the time of discharge. We aimed to identify common barriers and facilitators to shorter hospital stays and use the findings to inform implementation of the new ‘discharge to assess’ policy.

### 1.1 Conceptual framework

The theoretical framework for the study was ‘transitions theory’.^19^ In transitions theory, the relationship between transition conditions and outcomes has been supported and we thought that the model could translate to DToC as a transition. The authors therefore conceptualized DToC as a transitional state with opportunities for both positive outcomes as well as harm. Based on the tenets of the theory, we proposed that the key transition conditions (health service, social care and family or patient) could facilitate or constrain the transition out of hospital and influence outcomes including cost, prolonged hospital stay and physical and emotional impact on older people, as well as patient and family thoughts and feelings. These thoughts and feelings could in turn influence distal outcomes, such as seeking earlier discharge and better‐informed patient and family discharge decision‐making, leading ultimately to shorter hospital stays for older people. This study explores the transitional state of DToC and its impact on patient and family thoughts and feelings.

## 2 MATERIALS AND METHODS

This was a sequential QUAL–QUAL study^20^ with two components: A desk‐based review of older people's experiences of health or care services submitted to the Care Opinion online website,^21^ followed by telephone or video interviews with older people, and family members of older people, who had experienced a hospital stay within 12 months. Both components contributed equally to the study findings. Participants in both components were older people aged 75 years and older. Although globally the definition of older people has tended to be those aged 65 years or older, there is no clear rationale for this and there have been recent calls to redefine chronological old age. The Community Aging Research 75+ (CARE75+) study, from which interview participants were recruited for this study, recruits older people aged 75 years and older because at this age, important later‐life conditions are more prevalent and so it is more useful for studying epidemiological aspects in a time‐limited cohort.^22^ The authors therefore adopted this definition for eligible participants across all elements of the study to ensure consistency.

### 2.1 Desk‐based review of online care stories

Care Opinion is an online platform for sharing people's experiences of UK health and care services. We searched for submissions by older people (aged 75 years and older) or family members of older people detailing their experiences of recent hospital stays and/or discharge. Searches were conducted in November 2020 and were limited to those published since November 2018 to ensure relevance to contemporary care processes and pathways.

One author (H. S.) searched Care Opinion using keywords based on the inclusion criteria (Table 1). Two authors (C. G. and I. H.) removed duplicates and screened retrieved stories for inclusion (Table 2) and exported them into an Excel spreadsheet. C. G. and I. H. divided the stories equally between them and content‐analysed them using the conceptual framework for the study. Analysis began deductively—using conceptual framework constructs as initial codes—but concepts were also derived inductively as reading and analysis progressed.^23^

**Table 1:** Inclusion criteria for online care stories

Criteria	
Participants	Men and women aged 75 years and older.
Intervention	Hospitalized for any period of time for any condition or illness.
Context	Stories submitted from any country and region of the United Kingdom. Stories authored by older people, family members, caregivers or friends.
Outcomes	Thoughts, feelings or opinions about being in hospital, length of stay and discharge.
Language	Stories published in English.
Date limits	Published between November 2018 and November 2020.^a^

^a^ The database begins in 2005; we limited to 2018 onwards to ensure that the number of hits was manageable and the stories identified were relevant to current care processes and pathways.

**Table 2:** Search results and inclusion decisions

S. no.	Keywords	Limits	Hits	No. of duplicates removed	No. of stories screened	No. excluded	Total stories included in the analysis
1.	‘(elderly or old)^a^ and discharge’	11/2018	275	177	98	43	55
2.	‘(elderly or old)^a^ and hospital stay’	11/2018	27	12	15	3	12
3.	‘(elderly or old)^a^ and ward’	11/2018	1225	261	964	660	304
							371

^a^ All searches conducted on 9 November 2020.

One author (H. S.) periodically reviewed the codes and subcodes with C. G. and I. H., to resolve any issues and ensure coding consistency. After coding was finished, three authors (H. S., C. G., I. H.) reviewed the spreadsheet and regrouped coded data into categories of related concepts identified across the entire data set. Categories were further refined into six main themes (see the Supporting Information: File S1). These findings highlighted older people's and family members' positive and negative experiences of care and discharge, pertinent to the study research questions, and helped inform the topic guides used in telephone interviews with older people and family members.

### 2.2 Interviews with older people and family members

Data were collected in March 2021, when the United Kingdom was at COVID‐19 alert level 3 and government guidance allowed social contact indoors between members of a household only, which meant that hospital wards could not be accessed to interview older people during a hospital stay as planned or face to face in their own home following discharge. Therefore, telephone or video interviews were conducted depending on participant preference.

#### 2.2.1 Sampling

Maximum variation sampling was used based on factors known to influence older people's experiences of hospitalization such as gender, age and ethnicity.^10^ Using the inclusion criteria (Table 3), the initial sample included at least 10 purposefully sampled older people and family members with experience caring for an older relative during admission and after discharge. It was estimated that 3–5 more interviews would be required until no new ideas emerged—the stopping criterion.^24^ The sample was monitored for balance of inclusion criteria as recruited progressed. After each interview, the audio file and written notes were reviewed by H. S. to identify key issues and evaluate data saturation—which was achieved after 10 interviews with family members, and 6 with older people.

**Table 3:** Inclusion criteria for older people and family members

Criteria	
Participants	Men and women aged 75 years and older. Family members with experience of caring for a relative during admission and after discharge from hospital. English speaking. Able to give consent.
Intervention	Older people hospitalized for any period for any condition or illness in the last 12 months.
Context	A hospital stay is defined as being admitted and discharged from hospital for any length of time and for any reason.
Outcomes	Thoughts, feelings or opinions about being in hospital, length of stay and discharge.

#### 2.2.2 Recruitment

Older people and family members were recruited from a variety of sources:

1. The Care Opinion ‘research community’.^22^
2. NIHR‐funded CARE75+ study (a national cohort study including people aged 75 years and older recruited through general practices).^25^
3. Virtual ward at Bradford Hospitals NHS Foundation Trust (a home‐based intermediate care service, predominantly for older people with frailty operating a discharge to assess model).

Individuals were either identified by staff (Care Opinion's Chief Executive, CARE75+ programme manager or virtual ward healthcare staff) or self‐selected via publicity (CARE75+ newsletter). People who fulfilled the study eligibility criteria were then invited to participate via an emailed or mailed invitation letter, study information sheet and researcher contact details. Respondents making contact had the study aim, information and consent procedures explained and any questions answered, and an interview was arranged.

#### 2.2.3 Topic guides

Interview topic guides were based on the existing literature on older people's experiences of hospitalization and discharge,^26^, ^27^ a systematic review of qualitative research on older people's and relatives' experiences in acute care settings^28^ and content analysis of online care stories. Topics included circumstances of the hospital stay, benefits of being in hospital, difficulties faced in hospital, decisions made and feelings about discharge from hospital, discharge delays and views on the discharge to assess policy and shorter hospital stays. Interviews lasted approximately an hour and were digitally recorded.

#### 2.2.4 Consent and ethical considerations

Participants chose whether interviews were telephone or video based. Consent was taken before or at the time of the interview. Audio recordings, transcripts and study findings were rendered anonymous by pseudonym ID allocation. North West—Greater Manchester Central Research Ethics Committee approved the study (Ref: 20/NW/0478).

#### 2.2.5 Analysis

Audio recordings were transcribed verbatim, checked for accuracy and analysed using the ‘codebook’ approach to thematic analysis.^29^ One author read a selection of transcripts, listened to a selection of audio files and read notes taken after each interview, and based on this, identified key concepts and ideas in the data set and listed these as initial codes. Selected transcripts and the code list were shared with and reviewed by three other authors (L. N., A. D., D. F.), who suggested revisions or additional ideas. Revisions were mainly rewording of codes or merging similar codes. The revised code list was used to code all the transcripts in MaxQDA.^30^ Inductively derived codes were incorporated as the coding progressed. Coded data were collated and exported into matrices in MS Excel and further interrogated to collate related codes into themes. This process involved looking across the matrices to determine how different codes may combine into themes; some of the original codes were formed directly into themes and other codes were grouped together to form themes. At this point, the team discussed each preliminary theme to ensure that they told a coherent and meaningful story about the data. Preliminary themes were presented at professional meetings with geriatricians and applied health researchers working on patient safety and care transitions; some theme labels were revised following these discussions to ensure that they clearly conveyed what the theme was about. Supporting Information: File S1 shows how the team moved from codes to themes and subthemes.

## 3 RESULTS

### 3.1 Content analysis of online care stories

A total of 371 Care Opinion stories were content‐analysed; the majority (84%) were stories authored by family members or caregivers of older people and 13% were older people's stories (Table 4). Just over half the stories featured older women (53%) and 38% were about older men (in 9.2% of the stories, the gender was unknown and in 0.5%, more than one person was mentioned). In stories that mentioned age, 33% featured older people aged 85 years and older, 13% were aged 74–84 years and 4% featured those younger than 75 years (age was unknown in 49.6% of stories). The ethnicity of those submitting stories to the Care Opinion website was not recorded. Eighty‐six percent of the stories were submitted during the COVID‐19 pandemic.

**Table 4:** Characteristics of the included online care stories (*N* = 371)

Characteristics		*n* (%) *N* = 371
Gender		
Women		195 (52.6)
Men		140 (37.7)
More than one person in the story		2 (0.5)
Unknown		34 (9.2)
Age		
70–74 years		15 (4.0)
75–84 years		49 (13.2)
85+ years		123 (33.2)
Unknown		184 (49.6)
Location of the story		
Service or facility in the Y&H region (Bradford, Hull, Leeds, Sheffield, York)		23 (6.2)
Service or facility in other regions in England		276 (74.4)
Service or facility in Scotland		72 (19.4)
Story author		
Older person		49 (13.2)
Family member or caregiver		312 (84.1)
Friend		3 (0.8)
Unknown		7 (1.9)
Timing of hospital stay		
During the COVID‐19 pandemic (February 2019 onwards)		319 (86.0)
Before COVID‐19		52 (14.0)

Table 5 shows the findings, with themes and corresponding categories of data and the number of stories contributing data to each category to illustrate the magnitude of each theme. Three themes identified in the content analysis provide insight into the care environment and *how care was organized* for older people during a hospital stay, including deficiencies in care organization, how the discharge process was experienced and the extent of information and communication during a hospital stay. The other three themes outline *how care was experienced*, especially what it means to be old and in hospital, older people's unmet needs in hospital and family members' roles.

**Table 5:** Content analysis findings

Theme	Categories	Older people's stories (*N* = 49) *n* (%)	Family members' stories^a^ (*N* = 322) *n* (%)	Summary of the content of stories
Deficiencies in the organization of care	Care environment, transfers and moves	24 (49)	131 (41)	Bed moves and transfers of older relatives between hospitals, between wards, corridors to ward or aisle to ward and multiple bed moves while in hospital. Family members concerned about the number of times their relative had been ‘shifted around’, sometimes late at night. Transfers described as harmful or not ideal for elderly, frail people.
	Bed moves and ward transfers	10 (42)	66 (50)	
	**Organization of care**	**6 (12)**	**71 (22)**	Time taken to assess older people on admission, and older relatives being left ‘for hours on a trolley’ or in corridors before procedures. Staffing levels described as inadequate and wards as ‘seriously understaffed’; relatives noticed that staff were ‘overstretched’ and trying their best under difficult circumstances. Staff doing their best ‘despite the organization, not because of it’, in a system that seems ‘broken’ and a ‘service under constant pressure’.
	Time taken at points in the care pathway	0 (0)	5 (7)	
	Staffing levels	2 (33)	17 (24)	
	Management shortcomings	1 (17)	14 (20)	Having a dedicated nurse per patient meant that it was impossible to get help if that nurse was not available. Reliance on agency or bank staff who had no organizational knowledge and knew little about individual patients. Lack of continuity of care manifest as ‘different doctors and nurses almost every day’.
Disorganized discharge processes	Discharge processes	24 (49)	124 (39)	The discharge process was sometimes described as ‘faultless’ and ‘impressive’, with follow‐up appointments made, referral to community services in place and ‘detailed instructions’ for themselves and the GP. Negative experiences included being discharged with ‘no explanation’ or ‘no discharge letter’, no discussion of ‘how to cope when finally at home’ and a process that was ‘not straightforward’. Family described disorganized discharge processes; ‘chaotic’, with older people discharged without staff ‘realizing they needed to inform next of kin’, discharged at a different time than expected and family having received no notice of discharge or finding out ‘by accident’. Few older people who requested transport home as it was late at night, or they had alternative but were told by staff that it ‘was not their responsibility’ to arrange transport.
	Preparation for discharge	4 (17)	20 (16)	
	Swift discharge	2 (8)	18 (14)	
	Self‐discharge	2 (8)	12 (10)	
	Coordination and organization of discharge	6 (25)	25 (20)	
	Premature discharge	5 (21)	14 (11)	Some older people described still ‘feeling poorly’, being sent home ‘too early without any support in place’ and no discussion of ‘how to cope’ when at home; a common experience was of the hospital ‘getting people out of wards too soon’, when still ‘unwell and lacked capacity’. Older people referred to being discharged from hospital swiftly, in a matter of days, after surgery or operations. Two ‘self‐discharged’ due to the stress of being in hospital and ‘preferring to be at home’.
	Delays in discharge	5 (21)	35 (28)	Prolonged waits for ‘hours’ and sometimes until late at night for various procedures to happen before discharge including scans, physiotherapist assessment, consultant opinion or nurse assessment. Family recounted transport delays, relatives waiting hours for an ambulance to take them home, sometimes due to lack of communication with ambulance crews or poor communication between staff and family.
Communication is adequate but often deficient	Information and communication	29 (59)	209 (65)	Being ‘left in the dark’ and being ignored by staff when asking for information about their relative's condition; minority received ‘inconsistent’ or ‘conflicting’ information each time they called. Difficulty contacting the hospital or the ward by phone; having to call the hospital ‘repeatedly’; phones ‘frequently left unanswered’. ‘Grateful’ to doctors and nurses for taking time to answer questions or explain aspects of treatment and being kept updated even though staff ‘were very busy’. Older people said that staff were ‘informative’ and ‘friendly’, ‘helpful’, ‘happy to answer questions’ and explained procedures ‘quite clearly’ and in a ‘language I could understand’. Minority highlighted inadequate information and staff who lacked ‘common decency to speak to me’.
	Communication between staff	4 (14)	21 (10)	
	Communication with older person	18 (62)	33 (16)	
	Lack of communication with carer/family	7 (24)	155 (74)	
What it means to be an older person in hospital	Benefits of hospitalization	31 (63)	194 (60)	Being cared for meant feeling ‘reassured’, ‘relaxed’, ‘safe yet encouraged’. Attention from staff who were ‘caring’ and ‘compassionate’ and provided ‘constant supervision’ and ‘excellent care’. Feeling safe included staff who helped people overcome fears or nervousness about being in hospital and being ‘put at ease’. Family members highlighted staff empathy, compassion and patience, understanding of older people's needs, including those with dementia, memory loss or frailty and those with complex conditions. Also the relief they felt while older people were in hospital.
	Being cared for and feeling safe	21 (68)	84 (43)	
	Improved condition	10 (32)	57 (29)	
	Support with independence	0 (0)	4 (2)	
	Carer relief	0 (0)	49 (25)	
	Positive staff care	39 (80)	175 (54)	Older people described being treated with ‘respect’ and ‘as a friend’ despite their old age; also praised the professionalism and manner of staff when dealing with older people; examples included staff being ‘considerate of the elderly’ and recognizing their knowledge and previous experience. Relatives mentioned friendly, cheerful and positive staff and that ‘nothing was too much trouble’ despite the busy environment they worked in (all cadres of staff).
	Friendly and positive demeanour of staff	12 (31)	53 (30)	
	Staff patience and kindness	14 (36)	51 (29)	
	Being treated with dignity and respect	7 (18)	25 (14)	
	Negative staff care	9 (18)	76 (24)	Older people mentioned staff making assumptions that they were ‘not capable of making decisions’, arrogance or a lack of compassion among some staff towards older people who may be frightened or suffering with multiple conditions. Family described how older people had been ‘left alone’ or ‘abandoned’ for long periods; ignored by staff, requests for blankets, medication, food and water were ignored and ‘basic human needs’ were not met. Stories mentioned the lack of ‘respect’ for older patients and some staff lacked the ‘correct attitude to deal with the elderly’. Nurses and doctors were described as uninvolved, uncaring, cold, rude and unapproachable and doctors as ‘arrogant’ and ‘paternalistic’.
	Lack of staff compassion	1 (11)	10 (13)	
	Treated as unable to make decisions	4 (44)	2 (3)	
	Poor staff care and attitude	4 (44)	64 (84)	
Older people's unmet needs in hospital	Risks of hospitalization	19 (39)	189 (59)	Older people's physical or mobility needs often not met, including inaccurate assumptions about mobility, not helped to the toilet despite being unable to walk unaided and not helped to the car at discharge. Family mentioned staff not responding when the bedside buzzer was pressed or having to request help for their older relative because staff had not responded. Other times meals were left out of reach, or removed, instead of staff ‘encouraging or supporting’ older people to eat, particularly for patients with dementia.
	Unaccommodated needs	5 (26)	22 (12)	
	Feeling like a burden	3 (16)	9 (5)	Some older people felt like a ‘burden’ or a ‘nuisance’ to staff when asking staff for help. Family members treated as if they were ‘troublemakers’, ‘interfering’ or ‘an inconvenience’ when trying to act in their relative's best interests.
	Emotional stress	1 (5)	48 (25)	Older people preferred to be at home, felt ‘very anxious’ about not being told about their medical condition or anxiety was brought about by having to receive treatment in a certain way. The stress described by family members partly related to lack of information about their relative's condition; expressed as worry, disappointment and anxiety around not being able to ‘find anything out’ and how it was ‘stressful’ when you want information but cannot get it. Relatives mentioned feeling ‘fearful’ and ‘worried’ based on previous experience or stories they had heard about ‘how the elderly are treated in hospital’.
	Deterioration of condition	3 (16)	48 (25)	Some family members noticed ‘dramatic changes’ in their relatives, with visible deterioration in their appearance since being in hospital; others mentioned that mobility had declined ‘due to being stationary’ or left in bed for too long. Deterioration in older people with dementia was described in terms of increased agitation, distress and confusion.
	Inadequate environment	5 (26)	36 (19)	Inadequacies in hospital environment included unbearable ‘heat and lack of ventilation’ on the ward, helped by fans placed by beds but they ‘created white noise’; being made to go to bed at a certain time; and not being allowed to leave the ward to get food or drinks. Family talked about wards being ‘noisy’ or hectic and not places for older people who needed calmer surroundings; the ‘noise levels’ generated by visitors was ignored and the lack of privacy meant that conversations were overheard. The few stories that mentioned the impact of COVID‐19 centred on family not being allowed to accompany older people on admission or to visit; most understood that this was not possible.
	Impact of COVID‐19 on visiting	2 (10)	26 (14)	
Family members take on liaison and advocacy roles	Family and caregiver support	4 (8)	135 (42)	Family sometimes took on unanticipated liaison and advocacy roles for older relatives, including being relied upon for information, asked to ‘liaise between doctors’ on treatment decisions and negotiate with community services including the district nurse, mental health and safeguarding teams. Family members cared for and supported relatives on the ward. This ranged from washing, feeding and help getting to the bathroom, to assisting their relative in and out of bed and into chairs or wheelchairs; many mentioned that staff were too busy, overworked or were not allowed to help. Several family members said they felt that they had to be present on the ward to ensure that their relative was ‘treated with dignity’ and to get any information about their condition.
	Social contact with relatives	2 (50)	26 (19)	
	Involvement with health care	2 (50)	87 (64)	
	Waiting for services	0 (0)	17 (13)	
	Staff involvement	0 (0)	5 (4)	

^a^ Includes friends (*n* = 3) and unknown (*n* = 7).

### 3.2 Interviews with older people and family members

Ten family members and six older people were interviewed (Table 6). Most of those interviewed were over the age of 80 years and all were of White British origin. More men (62%) than women (38%) were interviewed. Family members tended to be sons or daughters (90%) of older people who had been in hospital. Older people had experienced hospital stays of varying lengths, with the majority admitted for four or more weeks (38%), and most experiencing the hospital stay during the COVID‐19 pandemic (69%).

**Table 6:** Characteristics of interview participants (*N* = 16)

Characteristics		*n* (%) *N* = 16
Participant type		
Older person		6 (38)
Family member		10 (62)
Age of older person		
75–80 years		1 (6)
81–85 years		6 (38)
86+ years		9 (56)
Gender of older person		
Women		6 (38)
Men		10 (62)
Family member relationship to older person		
Son or daughter		9 (90)
Niece or nephew		1 (10)
Length of hospital stay		
Up to a week		4 (25)
2–3 weeks		2 (12)
4 or more weeks		6 (38)
Unknown		4 (25)
Timing of hospital stay		
During the COVID‐19 pandemic		11 (69)
Before COVID‐19		1 (6)
Unknown		4 (25)
Recruitment source		
CARE75+		3 (19)
Care Opinion		10 (62)
Virtual ward at Bradford Royal Infirmary		3 (19)

Abbreviation: CARE75+, Community Aging Research 75+.

Six themes were generated (see the Supporting Information: File S2 for illustrative quotes).

#### 3.2.1 Older people and families appreciate the rationale for shorter hospital stays

This theme captures a range of beliefs and feelings about being in hospital expressed by older people and family members, including preferring to be at home, not wanting to be a burden on the health service and aspects of the hospital environment that were not conducive to recovery. These amounted to an appreciation of the need for shorter hospital stays (see the Supporting Information: File S2 for illustrative quotes).

3.2.1.1

**Family appreciates the rationale for shorter stays**

Family members explained how their older relative wanted to be at home rather than in hospital for longer than necessary. They talked about how their older relative liked being at home, explained that home was the right place for a quicker recovery and that an unfamiliar environment was not helpful. Family members also mentioned that getting patients home as soon as possible was understandable from a service perspective.

3.2.1.2

**Not wanting to be a burden**

Older people mentioned that that they were not keen on being in hospital longer than they needed to be and held strong views about being a ‘burden’ (sic) on the health service. They also talked about having to accept being in hospital and that it was the right place to get the care that was needed. Many older people also mentioned that they wanted to be at home, where they felt they would improve or get better quicker.

3.2.1.3

**Hospital as relief**

Several family members described feeling relieved when their older relative was admitted to hospital. Older people also talked about being ‘in the best place’ for the care they needed, being looked after and feeling like they were being taken care of.

3.2.1.4

**Boredom and lack of social interaction**

Some family members mentioned that their older relatives had described the helplessness of feeling ‘locked in’ the hospital environment, while others described the emotional impact of feeling lonely, especially not being able to see their spouse due to COVID‐19 restrictions. Boredom was frequently mentioned, and the lack of mental stimulation and opportunity for social interaction was a concern.

3.2.1.5

**Noise and ward environment not conducive to recovery**

The impact of the hospital environment featured frequently in accounts. A distinction was made between ‘mechanical noise’ from machines or transport of people and equipment up and down the ward and ‘people noise’ from loud conversations and people constantly coming and going. Related to this was the inability to sleep, due to being woken up by the noise and activity on the ward.

#### 3.2.2 Communication systems seem designed to fail

This theme encapsulates family members' and older people's struggle to obtain information, and their feelings of disappointment in the quality of interactions with staff. The apparent lack of information and poor communication were dominant in discussions with family and older people. Although the COVID‐19 pandemic was thought to have exacerbated communication problems, many family members implied that these were long‐standing issues and not just related to visiting restrictions and precautionary measures.

3.2.2.1

**Amount and quality of information provided by staff varied**

Many family members talked about the difficulties that they experienced trying to communicate with the hospital; some had to call several times to get through to the ward. When they did make contact, the level of communication seemed to vary by staff member. Others described the communication and interaction with staff as excellent. Examples included staff who kept family updated frequently, and doctors who took time to explain the condition and care process to older people.

3.2.2.2

**Important two‐way communication**

Several family members talked about the importance of a two‐way relationship with staff: building a relationship and having the right attitude helped create the right conditions for good communication. Older people too felt that building up a conversation with nurses was important and helped everything ‘go along very smoothly’.

3.2.2.3

**Poor communication undermines confidence in care**

A few family members felt that there was insufficient time for nursing staff to pay attention to communication. This created anxiety about their relative's treatment and undermined confidence in the care provided. There was a suggestion that doctors and nurses could benefit from a dedicated family liaison role, someone to communicate with relatives to free clinicians to concentrate on patient care. For some, the system was ‘almost designed to fail’ because of the inadequate communication.

3.2.2.4

**COVID‐19 exacerbated communication problems**

Several family members reported how the COVID‐19 restrictions on hospital visiting had made communication with their relative and staff difficult. Arrangements to facilitate face‐to‐face conversations were not in place or family‐provided iPads or mobile phones were met with resistance from staff. Older people also felt that COVID‐19 restrictions affected communication with family, that there was nothing to look forward to. Others said that they thought the inability to visit had affected their family members more than them. The predominant view was that COVID‐19 restrictions had exacerbated communication problems that existed before the pandemic.

3.2.2.5

**Feeling listened to facilitates care**

Several family members said that they felt listened to and staff worked with them to facilitate care of their relative. A few family members of older people with dementia and/or visual impairments highlighted that they wanted to support their relative but felt they were not always listened to or trusted by staff to provide that support.

3.2.2.6

**Communication between health and social care lacking**

Family members became aware of the relationship between the hospital staff and social care teams and reported that there seemed to be no link between the services and no evidence of them working together. There was a suggestion that a communications coordinator could facilitate communication between all parties and make discharge more efficient.

#### 3.2.3 Unwarranted variation and lack of confidence in care

This theme highlights the inconsistency in the care of older people while in hospital and how this affected confidence in the care provided. The theme also emphasizes the important difference that individual staff members can make at the time of discharge.

3.2.3.1

**Care varied depending on staff attitude and personality**

Care provided to older people while in hospital seemed to vary. This was experienced as inconsistency, and the general feeling was that care depended on staff capability and commitment. Some family members did encounter staff who they felt genuinely cared, but it seemed that this was the exception. The variation in care seemed to cause anxiety among family about the level of care received and how their relative was being treated.

3.2.3.2

**Family members lacked confidence in care**

Some family members appeared to lack confidence that staff were doing their best. This was linked to their experience of relatives not having their basic needs met and lack of age‐appropriate care for older people. Others felt that when staff were empathetic or took an interest, it was much easier to have confidence that their relative was receiving appropriate care.

3.2.3.3

**Individual staff members played critical roles**

Some family members mentioned a particular staff member who seemed to play a critical role in the discharge process. These were often nurses or social workers who knew the system, coordinated every step in the process and helped families to navigate social care. Often, family members described feeling fortunate to have had such help because it was not regarded as usual practice.

#### 3.2.4 Hospital discharge process caused frustration and anxiety

This theme captures how hospital discharge was experienced by older people and family members. It describes the frustration and anxiety that the process caused, as well as aspects that seemed to reassure patients and family and facilitate the transfer home.

3.2.4.1

**Discharge experienced as medical needs assessment**

For many family members, discharge of their older relative was experienced in terms of having medical needs assessed and ‘clinical sign off’. For some, the focus on being medically fit caused anxiety and family members worried about their relative being discharged too soon.

3.2.4.2

**Older people frustrated by discharge delays**

Several older people talked about discharge delays, resulting in waits of several hours or more. Waiting for medication to be prescribed and for transport to be arranged seemed to be the two main reasons for delays. Several described long waits for patient transport and ad hoc arrangements instead of a more organized system of allocating people to vehicles. Whatever the delay, older people were frustrated at waiting around for what felt like long periods of time, especially if they had got dressed, packed their bag and were ready to go.

3.2.4.3

**Quicker discharge due to COVID‐19**

For a few family members, not being able to see their older relative due to COVID‐19 restrictions heightened their anxiety about discharge because they had been unable to see them and assess for themselves if they were fit to go home. Some thought that the staff wanted discharge to be quicker and got the impression staff were quite anxious to discharge people.

3.2.4.4

**Family excluded from the discharge process**

There was a feeling among family members that they had not been involved in the discharge process. Some family members seemed relieved to not be involved, to not have the responsibility or because they had little to add to the decisions being made. Others were concerned about a lack of planning for ongoing care, including more information about the long‐term outlook, and support at home.

3.2.4.5

**Aspects of care that facilitated discharge**

Aspects of care that seemed to facilitate the discharge process included being discharged to a ‘virtual ward’ or bed‐based intermediate care (e.g., in a care home, community hospital or rehabilitation unit), which was described as reassuring by family and older people. Older people also mentioned the efficiency, availability and excellent care received from virtual ward staff. Family members mentioned other aspects that helped them and their relative prepare for discharge, including having information about how the discharge process worked.

#### 3.2.5 Family and older people unprepared for ongoing care needs

This theme highlights how unprepared family members were for the ongoing care needs of their older relative. This was manifest as not being told about the level of support that would be required, as well as feeling frustrated at not understanding how the health and social care systems worked. There was a perception that communication at transitions between secondary and primary/community care (e.g., district nurses and other therapy services) was where the system broke down.

3.2.5.1

**No help to navigate the social care system**

Family members expressed frustration at not being able to navigate the social care system in anticipation of their relative being discharged from hospital, especially patients not in receipt of government funding, who felt abandoned. For those being discharged to care homes, the lack of understanding about funding and how the system worked was even more acute. Others felt like they had not grasped or were not told about the level of support their relative might need once at home.

3.2.5.2

**Disjointed primary and community care**

Family members described how the care offered to their relative in the community was inadequate, to the extent that the system felt fractured. Some mentioned the lack of contact from GPs and having to follow up or arrange appointments themselves. Others experienced disjointed planning and management from hospital to primary care and being passed from department to department. Older people experienced inconsistency in district nurse availability and felt that staffing or funding levels were to blame.

#### 3.2.6 Factors affecting implementation of ‘discharge to assess’

This theme captures what older people and family members thought about the discharge to assess model. Many felt that it was a good idea, supported by their view that medical assessment should drive discharge decisions when an older person is able to manage basic functional activities, with additional assessment in their usual environment. Individualized predischarge assessment was thought to be important; one family member cautioned against viewing patients as Amazon parcels.

3.2.6.1

**Belief that discharge should be medically driven**

A dominant view among family members was that medical and functional assessment should drive discharge decisions. Some acknowledged that discharge assessment should consider more than physical fitness, such as assessment of psychological and emotional well‐being, to prevent people being sent home without support. Some considered discharge to assess a good idea but were not clear where people who still needed care but were unable to afford nursing care or had no family to take care of them would be discharged to. Older people seemed to think that the discharge to assess policy was a good idea and would prevent institutionalization.

3.2.6.2

**Barriers to implementing ‘discharge to assess’**

Older people and family members recognized potential patient safety risks with ‘discharge to assess’. An overriding concern was that without the right support in place before discharge, people would return to hospital with potentially more serious medical conditions. Another strong view related to the capacity of community services (e.g., district nurses) to manage older people discharged to be assessed. For some, the lack of state‐funded nursing homes combined with the demand from those without financial means was developing into a national crisis that required a solution.

3.2.6.3

**Facilitating ‘discharge to assess’**

Family members felt that managing their and their relative's expectations was important at discharge and that transparent communication would help facilitate discharge to assess. For example, setting an expected date for discharge was felt to be important and could be used as a positive incentive if they are ready to be discharged ahead of the expected date. Another view was that discharge to assess could succeed or fail based on transparent communication alone, especially if concerns and pre‐existing expectations are not addressed in advance of discharge.

## 4 DISCUSSION

Older people's and family members' thoughts and feelings about hospitalization and discharge revealed that the organization of care and how hospital care is experienced influence chances of effective transfer of care. Shared and sometimes negative conceptualizations of hospitalization and discharge and the context surrounding discharge decisions and planning can help or hinder shorter hospital stays.

### 4.1 Better transitions

**Figure 1:**
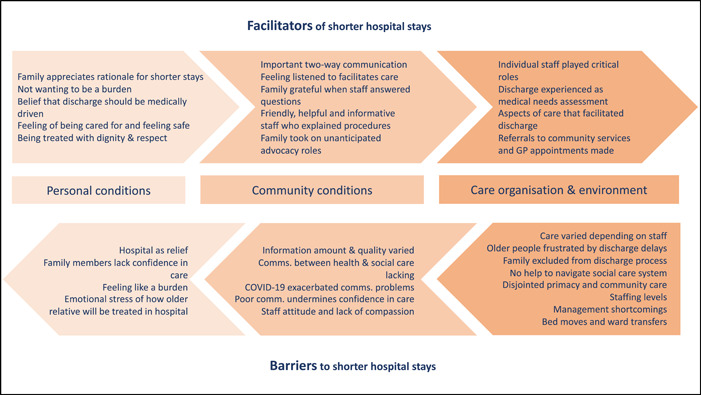
Barriers and facilitators to shorter hospital stays mapped onto transition conditions

Priorities for improving older people's transition out of hospital were determined by categorizing the findings into barriers to or facilitators of shorter hospital stays. These were then mapped onto the known ‘transition conditions’ outlined in transitions theory.^19^ Figure 1 shows the barriers and facilitators related to the care environment, personal and community conditions.

#### 4.1.1 Care organization and environment

Most barriers to shorter hospital stays seemed to relate to the organization and processes of care. These factors do not speak directly to the wider ‘societal’ conditions (norms and attitudes) outlined in transitions theory.^19^ However, the common decisions, norms and conditions within the healthcare environment can inhibit or facilitate transition out of hospital in a similar way. For example, older people were frustrated by the long waiting times and lack of organization at discharge. Suggestions included a more organized ‘system’ of allocating people to vehicles to take people home when they are discharged. Other research recommends standard protocols, online booking systems and real‐time information sharing between hospital departments to improve nonurgent patient transport.^31^

Discharge worked well when one staff member took charge of the process, coordinated assessments and facilitated communication between patients, family and staff and between the hospital and social care. However, this was not the norm, and those who experienced a single point of contact described it as ‘unusual’ and unexpected. While this might reflect that families lack information on the roles of staff in the discharge process, it also signals that a single point of contact was not always managing the process, and this caused some frustration. Age UK recommends discharge co‐ordinators to manage patient needs when a proposed discharge is likely to be complex.^2^ Designated discharge coordinators in mental health settings have also been shown to improve discharge and transitions of care.^32^

The hospital environment added to older people's stress and influenced preferences for recovery at home and early discharge. Hospital stressors have been characterized as an acquired generalized risk called ‘posthospital syndrome,’ which adversely affects recovery and can leave older people vulnerable to adverse events after discharge.^33^, ^34^ In our study, hospital stressors (e.g., noise levels, lack of ventilation, being woken up during the night) appeared to be widespread, but it is unlikely that they can be easily reduced in busy hospital environments. On the contrary, exposure to hospital stressors could be limited by shorter hospital stays or use of out‐of‐hospital services, which have been shown to lead to better outcomes in older people.^35^

#### 4.1.2 Personal conditions

According to transitions theory, when meanings and beliefs (personal conditions) are attached to a transition experience, they can positively or negatively frame expectations of a transition.^19^ In this case, older people believed that being discharged home to recover was best, they expected not to be a burden on services and this may have positively influenced their discharge expectations. In our small sample, we did not encounter any examples of patients wanting to stay in hospital longer or family members causing delays. Other research has identified similar perspectives, with older patients expressing the desire for timely discharge so long as they are physically well enough and have sufficient support at home.^36^

The findings also suggest that family and older people understand the rationale for shorter hospital stays and this can also prepare people and facilitate the transition out of hospital. A discharge to assess policy was deemed appropriate, if it paid adequate attention to patient safety, proper assessment, capacity of community services to manage discharged older people and transparent communication with family and older people. A recent trial of out‐of‐hospital services reported no increase in harm and reduced care home admissions in people discharged to a virtual ward service.^33^ Other research on discharge to assess with older adults with frailty found that they expected hospital staff to communicate clearly and plan with them instead of making assumptions about the care available at home.^37^ However, it is less clear how well patients' and family members' beliefs and experiences have been incorporated into the design of discharge to assess models, out‐of‐hospital services or other initiatives to improve delayed discharge.^38^

#### 4.1.3 Community conditions

The mapping also highlighted the importance of information, communication and support in the transition out of hospital. These factors are aligned with ‘community conditions’ in transitions theory.^19^ For example, problems with the amount of information provided by staff and difficulties in obtaining information as well as inadequate communication between health and social care teams were key barriers to discharge. A potential solution suggested by family members was a communications coordinator to allow medically qualified staff to concentrate on patient care. During the COVID‐19 pandemic, hospitals in the UK introduced Family Liaison Officers, and recent research supports the potential to standardize these roles.^39^ We recognize the potential of family liaison officers, with the caveat that clinical care and complex treatment plans would still need to be communicated by clinical staff.

Good two‐way communication is likely to create the right conditions for discharge, and our findings highlight the importance that older people and family attach to conversations with clinical staff. Potential solutions include providing older people and family members with a two‐way communication checklist of questions about discharge, which could help initiate conversations and help better prepare them.^40^ More strategic use of written information and predischarge meetings focused on discharge assessments and what to expect after discharge have also been shown to improve shared decision‐making in geriatric inpatient care.^41^

Deficient communication was a dominant finding and appeared so pervasive that it undermined confidence in the care provided. This appears to be a long‐standing issue, exacerbated by the immense pressures of the COVID‐19 pandemic. Other research, conducted before the pandemic, also suggests that lack of effective communication both between staff members^42^ and with the patient and family^43^ can delay discharge. Poor communication within healthcare organizations and with service users is a common and consistent cause of failure in NHS inquiries dating back to the 1960s.^44^ More needs to be done to uncover what drives poor communication, perhaps by analysing the behaviours and decisions that deter staff from providing respectful patient‐centred care and encourage poor care and communication.^45^ The coronavirus pandemic prompted rapid service modifications, changed the hospital environment and increased staff shortages due to redeployment or absence. This provided the perfect backdrop for job demands to outweigh job resources,^46^, ^47^ leading to decreased motivation and increased job stress and dissatisfaction. There was a perception among family members that some staff were ‘uncaring’; this is particularly concerning as it may be an indicator of apathy as staff reach burnout.

Lack of preparation for ongoing care needs of older people is another important barrier to transition from hospital. This was attributed to a lack of understanding about how the social care system worked and perceptions that district nurse and other community services were inadequate. That some described the situation as political and a budget fight between the NHS and social services is unsurprising after a decade of under‐funding in the social care sector.^48^ Disjointed planning and management from hospital to primary care perhaps reflects a failing of secondary care to put in place follow‐up appointments, and this could be resolved by ensuring that GP and outpatient referrals are in place before hospital discharge. Improved coordination between health professionals and integrated care models that minimize variation in care delivery are needed to ensure that older people's complex care needs can be met locally.^49^

The care stories highlighted that the family took on liaison and advocacy roles, acting as advocates, managers and guardians for older people during and after hospitalization. In the right circumstances, this kind of support could facilitate transition from hospital. These roles are documented elsewhere,^50^, ^51^ as is the negative impact of overburdened, anxious or stressed caregivers.^52^ In our study, the interview findings show that family members wanted to be involved in caring for their older relatives, but often felt excluded, not listened to or not trusted to provide support. Recent research recognizes that more collaborative involvement of caregivers as partners in care may improve hospital experience for older people and result in shorter stays.^42^, ^53^

Supporting Information: File S3 lists areas for improvement and suggested recommendations, based on our findings. These appear to be consistent with current policy‐level thinking on ways to reduce DToC. The recently published NHS guide to reducing long hospital stays contains several service recommendations,^54^ the ‘Where Best Next’ campaign offers principles for safe, appropriate and timely discharge^55^ and a recent scoping review identified initiatives that improve hospital discharge delays including tackling information sharing and altering how care is delivered.^30^

### 4.2 Research implications

The barriers and facilitators to shorter hospital stays identified in this study provide a framework for further research on implementation of discharge to assess in older people. For example, the framework could be used to assess existing discharge processes or to specify principles in the design of new discharge to assess models for older people. The suggested recommendations represent service adaptations that could be piloted to improve discharge to assess in older people.

More research is needed into the cultures and behaviours in NHS organizations that deter staff from providing high‐quality, safe, respectful, patient‐centred care. Research is needed to understand how behaviours become routine and normalized^56^, ^57^ and to identify the organizational cultures and behaviours that threaten care quality and safety.^58^ At a local level, trusts need to make the most of the range of methods available to collect feedback from patients and family on problems with delivery of care.^59^ There can sometimes be a gap between generating data on patient experience and making decisions to improve; this requires skills to analyse large amounts of diverse data and time to discuss and reflect on how to translate findings into action.^60^

### 4.3 Study limitations

This study has several limitations. First, the coronavirus pandemic and associated visitation limits undoubtedly impacted on patient and family experience of care and attitudes towards being in hospital, possibly influencing the findings. Lack of face‐to‐face contact during this period and the impersonal nature of telephone calls may also have impacted on rapport and trust between staff and families. Second, the authors were unable to recruit patients directly from hospital wards. This precluded collecting more immediate perceptions of time spent in hospital and decisions made during discharge. The authors were also unable to include a broader range of perspectives, especially those who stayed in hospital after being deemed medically fit. Third, stories submitted to Care Opinion may represent extremes of experience—those commenting on exceptional care and those who experienced poor care. Lastly, the patient and family sample identified as ‘White British,’ which limits the ‘representativeness’ of the sample. The authors had difficulty recruiting participants from other ethnic backgrounds. One of the main sources of participants was a sub‐group of the CARE75+ cohort, and acquiring assent from South Asian people in this group for involvement in substudies was difficult.^61^ The experiences of older people from different ethnic backgrounds and those who do not speak English may be different to those we report here. Recent qualitative research with a diverse sample of older adults found themes on experiences of discharge similar to those reported in this paper.^36^

## 5 CONCLUSION

This comprehensive account of older people's and family members' thoughts and feelings about being in hospital and the decisions made at the time of discharge provides a framework for implementing discharge to assess specifically and principles for new discharge models for older people generally. Recommendations represent service adaptations that could be piloted to improve organization of discharge, discharge preparation and knowledge and information and communication. The most prominent finding related to the quality and availability of information and communication provided to older people and family members during a hospital stay and at discharge. Communication is fundamental to patient‐centred care and even more important for managing interactions with older people and family in discharge models characterized by limited assessments and quicker discharge. Unless poor communication is addressed at the service level and through targeted patient information about what to expect in discharge assessments and after discharge, efforts to improve discharge of older people from hospital will be undermined.

## AUTHOR CONTRIBUTIONS

Helen Smith, Rebecca Lawton and Andrew Clegg conceived the study. Helen Smith had oversight of all stages of the research, collected data, led the data analysis and interpretation and wrote the manuscript. Chloe Grindey and Isabel Hague analysed online stories with support from Helen Smith and contributed to data interpretation. Louise Newbould, Lesley Brown, Andrew Clegg, Rebecca Lawton and Carl Thompson contributed to data interpretation and critically reviewed the draft manuscript. All authors approved the final version for submission.

## CONFLICT OF INTEREST

The authors declare no conflict of interest.

## Supporting information

Supplementary information.

Supplementary information.

Supplementary information.

## Data Availability

The data that support the findings of this study are available from the corresponding author upon reasonable request.
