# Supplementary material for: Reducing delayed transfer of care in older people: A qualitative study of barriers and facilitators to shorter hospital stays
Source: Health Expect. 2022 Oct 3;25(6):2628–44. doi: 10.1111/hex.13588 (PMC9700150; doi:10.1111/hex.13588)
Supplement: Supplementary file 1 — Supplementary information. [file HEX-25--s002.docx]

# Additional file 2.

Interview findings: illustrative quotes for each theme

| **Older people and families appreciate the rationale for shorter hospital stays** |
| --- |
| Family appreciates the rationale for shorter stays  “…he’s kind of a homebird. He likes being at home, he likes having his own comforts around him…” (CO-006_family)  “he felt he would recover quicker when he was at home, if he was, if he was going to recover, he’d be better at home” (CO_001_family)  “…if they say that the patient has fully recovered from their operation…and he’s back on his feet…you’d say, alright I hear that, let’s get him back out again then. Because I don’t think anyone really wants their relative to be in any longer than they have to be” (CO_006_family)  “…but there is that perception that hospitals are trying to shorten visits. And that’s understandable I suppose as an economic thing above anything else, I get that… I think there are a large number of people who are using hospitals, A&E and all the rest of it, as a place to go, rather than necessarily needing the services of the NHS. There’s a hell of a lot of wastage…certainly you know I do think that it is right and proper for people to be in for the length of time that they require to be in, not the amount of time that they feel that it’s nice to be in for a break…from an economic point of view there is merit in assessing people for being capable of going home or being capable of being moved on from a high-cost environment” (CO_006_family)  “…the risk of acquiring an infection while you’re in hospital significantly increases with your duration of stay, so, by limiting the amount of days in hospital, you reduce the risk of hospital acquired infections…” (CO_001_family)  Not wanting to be a burden  “I mean the main job had been done so there was no point in me lingering around was there?” (C75_004_older)  “Well, I think it was unnecessary…so, I just felt it, I was taking up their resources, you know, and I didn’t, didn’t need to” (C75_002_older)  “…you shouldn’t enjoy being there, really, if you do want to get fixed and get out again, that’s my feeling… I didn’t want to stay any longer than I had to” (C75_003_older)  “Yes, definitely. You feel more content when you’re at home, and I’m positive that you can, that your recovery will improve and get quicker by being in your own surroundings with people that you know” (VW_001_older)  “…I was sat in bed on the morning watching what was going on. There were people and they were far, far worse than me…And I’m looking at the fella in the bed next to me that was just laid there nothing, not talking, nothing, and I said to myself, please get me out” (VW_001_older)  Hospital as relief  “So, erm, I think, initially, for the family, there was the initial sense of relief knowing that he’s going to go in and, and get, you know, healthcare that, that he really needs” (CO-001_family)  “The benefit for me I’ve got to admit was great relief yeah less so my mother…” (CO_004_family)  “Yeah…it was an enormous relief that she went into the hands of professionals…and the relief was that she’s in the best possible hands then, so that was good. The whole thing was quite a relief for my dad…so he got some respite, yeah, that’s probably the best way to describe it” (CO_005_family)  “I was only in a short time but being there and knowing I was being looked after by people who knew what they were doing eased my feelings greatly. You felt I’m not neglected, I’m not stuck on my own…all I’ve got to do if I need anything is to press a button if I’m worried at all, and someone will come…a feeling of confidence” (VW_001_older)  “…but there was no, I didn’t worry about any of that, I knew I was in the best place for the job to be done” (C75_003_older)  Boredom and lack of social interaction  “Exactly, and I think if, if, if, despite your age, you have all your faculties, erm, I think it, it, it exaggerates…the fact that you’re in an environment that you can’t control and it gives you a feeling of helplessness, and that’s the way that my dad described it…he said you’re locked in an environment where you can’t do the things that you would naturally do and you can’t remove yourself, so there’s a sense of frustration” (CO-001_family)  “So, I think he definitely felt a bit lonely…so I think that affected him, you know…he was pining for my mum and that was having a really negative effect on his wellbeing….” (CO_010_family)  “…because she used to get terribly bored because she had nothing to do. So, they, they, you know…kept trying to find things that would interest her, you know, and keep her brain ticking over” (C75_001_family)  “They do need people to stimulate them, and the older they are, because they are probably inactive physically, mentally they will need to keep aware, is what it is… erm, and in the hospital there wasn’t really enough for her; she wanted to do things and, well, you can’t in hospital. Because everybody wants to do it for you, so you don’t fall” (C75_001_family)  Noise and ward environment not conducive to recovery  “Noise, the noise was very big, one of the biggest things I remember was the noise. Some of it was mechanical noise, some of it was people just talking across the width of the ward to each other, shouting at each other, erm, as conversation… erm, the noise was terrible, not so bad, maybe, at night” (C75_003_older)  “I think generally there is that issue of, you know, just noise levels and other people being, because if you’re in a sort of general ward, that you get the other people not sleeping properly, that’s the thing” (CO_009_family)  “I found it difficult sleeping, I always do in hospital because there’s always something moving, something ticking, something ringing” (C75_004_older)  “No, it’s not conducive to trying to get well…it’s, it’s like sitting in the centre of [CITY] with buses going by, it’s almost equivalent of…it was very, very noisy” (C75_003_older)  “…he was in a four bedded room and he said it was quite difficult to, to, to get, to get a good restful night’s sleep because of the constant interaction with nursing staff and everything else…” (CO_001_family)  “Oh, yeah, they woke me up at stupid times. They woke me up to give me a tablet to send me back to sleep again. You know, absolutely stupid things they do. But they’re just following protocol…but that were ridiculous” (VW_003_older) |

| **Communications systems seem designed to fail** |
| --- |
| Information amount and quality provided by staff varied  “No, there was no communication with the hospital at all, I mean they, at no point did they ring me or update me…but even if you could get someone to pick-up the phone, getting hold of some who actually knew him was virtually impossible and then getting any sense out of them was difficult” (CO_003_family)  “And I wasn’t pestering, I restricted myself to one conversation a day because I knew that people were busy and I didn’t think that that was unreasonable, so you know…but it would often take me ten attempts to get to talk to somebody” (CO_005_family)  “The annoying thing…we took it in turns to phone, but we found we had to take it in turns over the course of a day because we couldn’t get through. And then when we did get through the information was, basically, off a sheet. Erm, bog-standard answers…So that wasn’t great because you couldn’t tell if you were being told what they wanted you to hear, or what was true” (CO_006_family)  “In terms of the communication, we would phone the ward every day, every morning, erm, to get an update on, on his progress…erm, being blunt about that, that, that varied significantly from individual to individual, erm, and I, I think that’s something that could be improved” (CO_001_family)  “Yes, they told me that the consultant would be round after 8 o’clock…he came and sat on the bed and had a good talk and showed me the x-rays, which was brilliant” (VW_001_older)  Important two-way communication  “We managed, it was a two-way relationship, I felt, you know it wasn't just about me phoning about mom yeah it's you've got to work with the care profession, you know, that's all I can say they were absolutely wonderful” (CO_004_family)  “Communication is really, really is key, I would say that is the primary. If you've got the comms right, you can move things, progress things yeah” (CO_004_family)  “Yeah. I think, in a way, if you start saying, ‘I must know what’s going to happen to, and I must know…’ it doesn’t do any good...if you ask, ‘would it be possible…?’ Then you know, it’s the attitude I think, in a lot of cases, erm, that people have towards the staff” (C75_001_family)  “…very often the nurses would say “oh you come from [town], you know, I’ve got so-and-so in [town]” and you build up a conversation which helped it then go along very smoothly” (C75_004_older)  Poor communication undermines confidence in care  “Erm, I think the communication is something that, that is really key, and I, I wouldn’t blame the nursing staff per se for that, but I don’t think there’s sufficient time during the nursing handover to, to really pay the attention to detail that’s necessary” (CO_001_family)  “…there are one or two of the nursing staff that are excellent in that level of communication and there are others that, that, to be honest with you, it was quite clear that…they were there to, to earn a wage rather than there as a vocation, erm, and that’s disappointing, when you get that, because then raises your anxiety, that, is my dad getting the right level of care here, if this is the way that they communicate his condition” (CO_001_family)  “Well you could never speak to anybody that appeared to have any kind of continuity in the story…in fact relatively senior clinician told me they never have time to read the notes anyway…so to then have a discussion with that person with any confidence about, you know, their insight was, I just had no, there wasn’t any” (CO_005_family)  “…a communications focal point in each ward. So that the clinicians can actually get on with what they’re doing…introduce a new role of communications coordinator for each ward so that there is no distraction to the clinical services” (CO_009_family)  “but institutionally it’s broken…it’s almost designed to fail, the organisation, because it’s so chopped up that the success of one doesn’t depend on the success of the other…they’re not all in together…that concept seems to be totally foreign” (CO_005_family)  COVID-19 exacerbated communication problems  “…when dad went into hospital prior to Covid I spent 24 hours a day with him because I knew the difference it made and I could do it…but I didn’t realise until Covid how much difference that had made….” (CO_003_family)  “My theory is that in normal times the onus has always been on relatives, friends to follow-up, and the NHS has been able to rely upon that…now when Covid came it was impossible for friends and relatives to follow up...but the people in the system forgot to put in place any alternative communication protocol, I think they were overwhelmed…” (CO_005_family)  “We were told there was there was no time and we were told the technology wasn't available. I offered to take an ipad and leave at the hospital, so staff members could contact us when…we were told that they didn't have time and, yes, she could have if she could operate it herself… there's no way, given B’s situation, that is not going to happen…it was not a priority” (CO_007_family)  “Oh, that’s a downer, that’s, it would have been much, much better if you’d had visitors, you know, it would be summat to look forward to…that’s what I feel about that” (VW_003_older)  Feeling listened to facilitates care  “yeah yeah and I, for one, I felt someone was listening to us” (CO_004_family)  “Yeah, absolutely, absolutely, they were... I mean…they were interested in the things that I had to say about it and what I felt about it and that kind of thing and that did have an impact on whatever they decided to do eventually so yeah, I do, did feel that” (CO_010_family)  “My experience with B was not that she was refusing to drink, but she needed somebody who understood dementia…nobody asked me about anything about how to help B communicate…I had to keep saying that time and time and time again…hearing is very different from listening…and I felt that some of the staff I spoke to they never acknowledged what they had heard” (CO_007_family)  “there was no structure within which I could contribute my knowledge of him to enable his better care…sometimes they just don’t want it…over the previous nine years I have encountered all sorts of attitudes from staff telling me that they know their job and don’t tell them what to do and I’m not telling them what to do, I’m trying to share my knowledge of what works with my dad” (CO_003_family)  “And yeah and I think, especially if you know an elderly person has dementia, to give them if possible that right to let them have that support that they need beside them because you wouldn't take a wheelchair away from someone in a wheelchair. I mean why would you take away the human support that makes their life, the quality of life so good really” (CO_007_family)  Communication between health and social care lacking  “So at that point that made me feel well they obviously don’t get on with each other, there seems to be little respect, so I wonder where the respect for everybody else is, and that kind of misguided humour there, and cynicism for a relative that doesn’t have a clue what’s going on, is not helpful” (CO-005_family)  “I don’t think it’s necessarily against each other, I wouldn’t go that far, but I don’t think there’s any incentive for them to work together” (CO_005_family)  “And this was the problem all along, was the lack of communication between…[hospital] and [care home]. I fear that she deteriorated because of that, erm, more than anything else. I don’t, I know how busy they are, and I know how difficult it is…all the way along you felt like everybody wasn’t getting communication from one to another…” (C75_001_family)  “I mean, at one point we discovered that they hadn’t talked with the Social Worker, so we just then contacted the Social Worker, and said, “Look, you need to get involved. They’re having discharge meetings, here’s your contact”, and putting the contacts together…so you could introduce, probably, some role linking that in with that coordinating, communication coordinator, because that, they could tie in with all the relevant parties regarding discharge as well, couldn’t they, to make it more efficient” (CO_009_family) |

| **Unwarranted variation and lack of confidence in care** |
| --- |
| Care varied depending on staff attitude and personality  “It wasn't consistent it wasn't reliable it felt as if you were relying on personality. Yeah” (CO_007_family)  “He, he felt that some of the staff were exemplary and that, that they couldn’t do enough for you… Erm, but, for the ones, the, the difficulty is you could have ten excellent nurses providing excellent care and one nurse or two nurses that were really quite poor” (CO_001_family)  “Nobody goes into a caring profession that’s poorly paid and very demanding, mostly they don’t do it unless they really care about people’s welfare, but somewhere along the line because their goodwill is abused, because the way that the system is politically organised, what they do in caring is not valued…you can trace this back politically, it goes, it really is top-down, it’s structural, so there is a very small thread of people who go into it for very perverse reasons but the majority of people go in for good reasons, to try and do a good job and they get worn down” (CO_003_family)  “I think you know, in all walks of life we’ve got people with different aptitudes and different levels of commitment and capability and that’s just, you know…one or two that were just absolutely wonderful, and we had one or two that you know, couldn’t wait to get away, but and I get that individual variation, you always have that” (CO_005_family)  “most of the people in the hospital are great and they do everything to help you, but there’s the odd person who you know, just isn’t suited really to be caring for people [laughter], you know” (CO_002_family)  “And I am impressed with the people who maintain their humanity, who do bring me a cup of tea at three in the morning when I’ve been sat there with him all day you know, and they are still there, those people are still there” (CO_003_family)  Family members lacked confidence in care  “And quite often they're even the addressing them in a way that isn't appropriate for the older person either. You know, sometimes an older person likes to be called Mrs Roberts rather pet or love. That's called culture, the culture is not there” (CO_007_family)  “But if you take a bit of interest in someone you can strengthen that bond, and you can work with them…I just wanted to make sure mom was getting the care, but if there was anything wrong they could tell me…if there was anything I needed to address” (CO_004_family)  “you know that when I spoke to staff who just took her experience at face value…so where there was empathy and trust, that, you know, it made such a difference, it was a relief yeah and it was very difficult yeah” (CO_007_family)  “If you feel like somebody's not looking after him properly, but we had the benefit of seeing how well they were looking after him and then confidence in their, you know, ability to do that and their connectedness to him, you know, their concern for him and that kind of thing so that made it much easier to leave him” (CO_010_family)  Individual staff members played critical roles  “I have to say that, that in terms of the discharge process, erm, I, I was very impressed. Erm, I felt that the charge nurse, he was exceptional…his bedside manner was, was very good, he sat down, and he spoke to my dad, he explained that he’d been nursing him for a few days now and that he felt he got to know him…then he then coordinated the Hospital at Home visit, and that was quite reassuring for the family…that was all set up around about his discharge time, which I, I have to say, I think was exemplary, and it, that, to me, would be the model that you would use, where his home needs were assessed, they were dealt with his medical care, er, his medication and everything else was, was put in place for him being discharged and, er, the follow-up visits at home from, from, you know, trained staff that, that were, would have his medical history, that, for me, is the ideal model for someone that’s actually being discharged from hospital” (CO_001_family)  “…and it’s probably down to that one individual driving it forward, but I, I would say it was exemplary because I think, erm, he, he made a very good summary of my dad’s situation, he tried to do what he believed was the right thing, but importantly, he recognised that, that the right thing on this occasion was what my dad felt needed to happen, and he took the extra steps, if you like, to ensure that, that he got the best of a difficult situation, and I, and I think that, to me, is, is, is the, the perfect, it, it’s the perfect scenario” (CO_001_family)  “…I was allocated another social worker…I got a young, erm, girl, and she was fantastic. She said, I know your mum’s self-funding, but if you want any help, I will do it for you. So, I rang her, and she said, ‘look, save your ringing around nursing homes, I will find one for you,’ and she did… they don’t have to be a social worker, but if they know the system, and tell you, you know, what you need to do. But trying to find out everything on your own is so, so difficult” (C75_001_family)  “It was the OT and she was fantastic, I have to say…asking about his baseline and that sort of thing and she was telling me what was happening with the physio and what was the house like at home and all that kind of stuff…and she kept in touch with me about the timing of it and how it was going to work and what time he would maybe get home…she co-ordinated with all the other people and she ordered him everything he could possibly need now or might need in the future…I did say to her, if this discharge is unsuccessful it will not because you haven't been attentive enough…honestly, I don't know if I would ever know anybody that would experience that in the way that we did because I think it is unusual. I know it's sad to say but I think it is unusual” (CO_010_family) |

| **Hospital discharge process caused frustration and anxiety** |
| --- |
| Discharge experienced as medical needs assessment  “it sounds exactly like what happened when mum came out of [hospital], because there was this emphasis on her being medically passed if you like by a doctor of a certain rank to, and then go home, so it sounds like that’s what it was…that’s what they described…it’s an absence of found problems, not a positive assessment of fitness, and I think that might be the fundamental issue” (CO_005_family)  “And as it turned out, you know…certainly their intention seems to have been, you know, getting him as well as they possibly could, and they carried that out. So, I can't criticise it at all because, certainly from the OT's perspective, she seemed to know him and understand what he needed and all that, so yeah. But I think it was the right thing [to discharge him] even although I had some concerns about it” (CO_010_family)  “Yeah. I think looking back on it, erm, at the time we weren’t entirely sure how he was going to cope. Because we’d been up, I could see how he was walking, erm, he was in bed basically all the time I was up there, or he would be sitting on a chair beside the bed. He would go to the toilet a few times and he would need help across to the toilet. So, you then think, how’s that going to work if he’s at home because he lives on his own” (CO_006_family)  “Was she's medically fit, the message from the medics. And that that may be so, yeah maybe so but in terms of her ability to understand things, she's not she's not yet she's not…we were unsure at that point whether or not she would be fit enough to go home. When I was communicating with her on the phone I, because I know her pretty well my instinct was saying she's not, you're not actually fit at all to come home (CO_007_family)  Older people frustrated by discharge delays  “The only waiting time that I got a bit, not annoyed at, but agitated at was having to wait for medication to you know, to come to let me go, to get rid of me kind of thing. Although, I didn’t have any medication to bring home, but it was waiting for the doctor to come to sign the discharge sheets and say, “go on, away you go” (VW_001_older)  “Well, you’re ready to go home and you pack your stuff up. And you’re there for hours. You know, if I knew that, I packed my stuff and then we’re off, you see, you’ve got so long to wait for medication and stuff that, they’re changing your tablets and stuff like that in the hospital and knocking tablets off and giving you a new type of tablet…waiting for the medication coming from the pharmacy in the hospital. Or they haven’t got it and they’re waiting for it coming in, they’ve had to order it, do you know what I mean? They give you some excuse, well, there is an excuse, I don’t know, but there might not be. But you don’t know, you just take their word for it ” (VW_003_older)  “So when it came to getting home, it's a case of that's one of the big problem I have…to get you away from hospital it's hell because you're waiting and waiting and waiting for somebody to come and pick you up. If you're like me you're in a wheelchair…you're just waiting for people to come and pick you up. You want to go home, you can go home, nobody's said you can't, but you can't get there because there's nobody to take you home” (VW_002_older)  “I know people are busy but surely there should be some system where people, when are people are ready to go home they can get home…to have somebody there that does take people home rather than wait for people who are passing in their way, you know…they don't seem to think about that” (VW_002_older)  “No, the only thing, I would have preferred it if patient transport could have got to me a bit earlier. I’d got to wait I’d say about 2 hours or something like that when they said, “you’re going home, I’ll ring patient transport”…I wouldn’t complain about patient transport at all because I think they do a terrific job, and again, it's not just me that they’re looking after, they've got hundreds of other people to sort out and get home and whatnot, so. But if that time from saying “yes, you are discharged, you’ve got everything”, if the transport could be there in say half an hour, that would be a lot better than waiting two and a half” (VW_001_older)  Quicker discharge due to COVID-19  “You know, we would say to them, “Look, how is Malcolm feeling about this?”, because you can’t have that conversation because you’re not there because of Covid regulations” (CO_009_family)  “I was worried about him coming home because he had delirium, you know, so he was completely…they let my mum and I in to see him every day like, but that didn't happen once he went to [hospital]. So we, it was harder to assess whether he was ready to come home at that point or not because we weren't seeing him and that was distressing my mum greatly” (CO_010_family)  “Yeah, so the time in July she was in the hospital in 3 days, I think it was partly because of the Covid situation that they wanted it to be quick…it was as quick, it had to be…I think it was quicker than normal because of the pandemic situation” (CO_002_family)  “I think it becomes, it’s just difficult to make it personal, I think, for them. You know, you’ve got the impression, I think, because of Covid, particularly, they’re quite anxious to get people out of the way. So, it happened very quickly and very efficiently. But possibly a little bit impersonally” (CO_009_family)  “When we were told that she was coming home and her condition was described to us…I was very worried and a little bit shocked and very worried that she wasn’t well enough to come home…well I was nervous and anxious, that’s for sure, and but maybe that’s you know, maybe that’s just absolutely natural, that’s just what happens, as it turned out I think she was fine when she got home…” (CO_005_family)  Family excluded from discharge process  “I don't feel we were involved to any extent I think we were we were being told what was going to happen. Yeah, I don't think we were actually involved at all…I would like to have had a proper meeting with, you know, a member of staff. Yeah, preferably face to face rather than on the phone. Because it's easier this way, and so I would like to have a meeting about that. Like to have had more information and you know, be aware of what, you know, what the outlook was for her. Would like to have a plan, there was no plan at all” (CO_007_family)  “I don’t know. Because the hospital set it up, I wasn’t involved except that I got two or three phone calls about what they were going to do. But what they actually said to my mum I don’t know” (C75_001_family)  “To be quite honest, I was relieved as it was taken out my hands and the responsibility yeah um and also mom being brought home by patient transport because they could manage her and lift her which I couldn't do” (CO_004_family)  “…so I knew in advance what they were doing, that they had an idea of who was involved in that process from their side, erm, and the mechanics of how it would work. So, I felt that it wasn’t my decision to make…but I think to the extent that it was helpful for me to be involved I was involved. Again, it was more about the communication of that, and the process, rather than sitting down at a table and discussing it all because I don’t have very much to add to that decision” (CO_006_family)  Aspects of care that facilitated discharge  “Yeah, the virtual ward. The staff on them, I mean, they put themselves out big style. They’ll come at nine o’clock at night and bring you some tablet or something that you need to get through the night, you know” (VW_003_older)  “They did come to me for two or three, three or four days afterwards, you know, to check on me each day which I think was really good actually, yeah. Yeah, yes, and they're providing me with one or two things that were needed, that's a commode and a bottle and yes, yes, I think that's a good idea, is that virtual ward thing” (VW_002_older)  “…and then you’ve got the virtual ward, which I can’t praise highly enough. The district nurse situation, it’s just a little bit different to me… whether they’re short-staffed, well I mean they’re obviously going to be under pressure, but they, at times they haven’t visited when I thought they should’ve done. It’s sort of taken another week longer, but I wasn’t desperate anyway. But the Gold Line is brilliant, and the virtual ward is exceptional” (VW_001_older) |

| **Family and older people unprepared for ongoing care needs** |
| --- |
| No help to navigate social care system  “…then you’ve got this budget fight between the NHS and Social Services and continuing healthcare has got a six-month limit on it and it’s very political. It’s very constrained, so you know, these people are trying, and I had no understanding about how the funding for care homes works or you know, none of that, I know now but I think the whole structure is just incredibly cack-handed…” (CO_003_family)  “…and all of a sudden I need help for my 89-year-old mother who has always stood on her own two feet. And now because we’re not on benefit, we’re not in their system, we get hee-haw, you know. If you’re on the cadge you get… your rent paid, you get everything given to you for nothing, but if you’ve stood on your own feet and paid your bills you get nothing. You get absolutely nothing” (CO_002_family)  “We were not, I mean particularly then you know that transition, there was no regard given to if B could be properly supported when she came home…but to be honest, the kind of advice that the carers were able to give us we didn't need that kind of advice it wasn't that kind of advice we needed yeah…we felt isolated and we felt we felt the family had been put at risk as well…what she needed was a thorough assessment and a plan for when she was discharged” (CO_007_family)  “…she did more than social services would have approved of, really, erm, if they’d have found out what she was doing. She wasn’t doing anything that was in her job description, it’s just that, my mum being self-funding, nobody on the NHS-government side of it want to know” (C75_001_family)  “I think it was probably two things that I didn’t feel that, either were told or I took in properly. The first one was the level of equipment they would give us for him coming home…And I think the other thing was, probably not necessarily knowing what the next stages were. And I suspect that part of that is probably that they didn’t know themselves what the next stages would be…What we didn’t know was, erm, would he go to his GP to assess how he was getting on, would the occupational therapist assess, would the physio assess, or would he be coming back into the hospital” (CO_006_family)  Disjointed primary and community care  “and so I could I feel for the staff because I could see that they were frustrated that they weren't able to be able to offer the level of care out in the community, that they would have liked to offer. It felt as if everything was very fractured in the system” (CO_007_family)  “…this is where the system falls down is the follow up GP care. That was where we felt really let down, I contacted the surgery took the paperwork along popped it through the door….So that's where I think when it goes back, you go back home, the system falls yeah so badly…because I felt the GP or practice nurse could have maybe followed up rather than myself just to check if it's only I knew something was wrong with one of her medications that I chased that up…” (CO_004_family)  “And when you said well you know, we don’t have to deal with these problems, this is all new, the answers were along the lines of…we’ve asked for a care package…so when I said then said well is that going to happen, you know, or is she just going to turn up on the doorstep and the care package doesn’t come, it’s well we’ve asked, it’s somebody else to deal with it, and it was so disjointed, I mean my impression was that each NHS department just wanted to hand off to another” (CO_005_family)  “When we were told she was incontinent, you know, I said well how do we deal with that, and the people at hospital said, well you have to phone, I can’t remember the exact order, it’s in the story, it’s like you have to phone the incontinence and something or other team, okay I’ll phone them, so I did that…and their answer was, well we’re not dealing with that, that’s now done by the GP…so you phone the GP and the GP says, well we’re not dealing with that, that’s now done by the district nurse, that is at such and such, so I was handed off four times in about two days….it’d be helpful if you medics came to a recommendation and didn’t recommend against each other, but the GP side and hospital side they might as well have been on different planets” (CO_005_family)  “And it took me 3 weeks sorry, and I went through seven departments. I phoned-up, “oh, it’s nothing to do with us, you need to go to here”. Went there and I got that, seven different departments I went through to get an answer at all, and I eventually did get people who helped me, and I got a physio, which took 7 weeks from my mother getting out til a physio coming” (CO_002_family)  “Well it all boils down to staffing levels, doesn’t it. It comes down to what bodies the various organisations have to look after us. I mean, it would be good from my point of view if a district nurse called every other day…there’s going to be more and more cuts coming on and they'll all be cuts in the wrong place from my point of view. Elderly care is really critical, and money should be put into it to help people look after the elderly. Because unfortunately we are living longer and our health deteriorates the older that we get. But it all boils down to money and staffing doesn't it?” (VW_001_older) |

| **Factors affecting implementation of ‘Discharge to Assess’** |
| --- |
| Belief that discharge should be medically driven  “I think, erm, I think the overriding criteria for that should be the medical assessment and that as soon as the patient is ready to be discharged from hospital, medically, then they should be… the overriding control should be on the medical assessment, and the medical assessment could be both in physical health but also mental health… there’ll be some people that, medically, are probably fit to be discharged but, psychologically, you know, need, need, maybe, a bit more reassurance and a bit more confidence, and there’s others, like my dad, who, psychologically, was fit to be discharged but, medically, you know, probably a bit premature….and I think the overriding control in that should really be the medical grounds.” (CO_001_family)  “…you’re not, you know, not treating packages for dispatch, you know, you’re not a delivery company, you’re not an Amazon worker… taking stuff of a conveyor belt and putting into the back of a van and saying okay, that’s it, done…if your parcel is delayed by a day, hey, it’s not the end of the world, if your patient’s discharged and they’re not medically fit to be discharged or indeed don’t get the correct follow-up care, it is life or death…making a mistake with somebody’s health is, is a disaster” (CO_001_family)  “I think that’s right but I suppose the other side of that coin is, if it’s a mental or an emotional thing, is an NHS bed in a hospital the right place for that person, or should there be somewhere else that they can go…so I do feel that the medical thing has to be the driver to it, and I do accept that there has to be assessment of other things, emotional and mental support. But emotional support doesn’t necessarily mean you’re taking up an NHS bed” (CO_006_family)  “I would think when someone has been clinically declared okay, we’ve done all we can…there’s no point in you being here, get yourself home. Because I would think 99% of us will improve at home better than being sat in a hospital bed or a chair watching other people far more ill than we are, it could get depressing” (VW_001_older)  “…what I’ve actually noticed with people being in a while etc and they get almost institutionalised. It’s nice being there and being looked after and they want to stay a bit longer and I think, but I think the policy is good because, you know, once you’ve finished all the nursing care you need and you’ve had all nursing treatment you need and it can be handled at home then that’s good, it will empty the hospital quickly and make room for another patient” (C75_004_older)  “We’re living longer…we are going to be faced with this more and more and more now. And I don’t think we’re set up, as a nation, to have the financial support from the Government to put people into old folks homes. So therefore, the NHS is, I think, undeniably getting completely blocked up. Because what do you do with these people who are very ill, too ill to be looked after, they can’t afford to go into private nursing care, what the hell do you do with them?“ (CO_009_family)  Barriers to implementing ‘Discharge to Assess’  “I think it's awful. I really do I think it's got so many holes in it, you know what we've just spoken about. I can see so many pitfalls there for the older people and families who support them. I, and I could see how it could actually end up putting more strain on the system because you may actually end up with people back in hospital who wouldn’t have been in hospital otherwise” (CO_007_family)  “…the risk, you know, we, we all need to have a, an element of risk management in what we do, I suppose, and if you were to do a risk analysis of that…erm, if a patient is discharged to home and then that assessment is made, erm, stable door horse bolted kind of springs to mind…what happens if that patient is in the home environment and the assessment is that they should really be in hospital?” (CO_001_family)  “I'm a great believer yes, as soon as you're on the mend home is the best place but you've got to have the support to help you. And if you've not got that at home, even with district nurses or other carers it could be quite dangerous. If you're discharged with a stick jeez, you know what the older generation’s like” 9CO_004_family)  “What happens if they say well medically fit to go home but you know, the person has gone from, let’s say living on her own, when they went into hospital, and they deteriorated, that left them really not safe to be at home. You know, who is saying well this can’t just happen tomorrow afternoon at four o’clock, because it doesn’t work, that would be my, you know, who’s looking at the overall picture” (CO_005_family)  “You cannot turn round and say, “It’s not our problem, we need the beds”, when there isn’t a solution. You need to find the solution to the problem….you’ve almost got to say, “Well, sorry, but whose responsibility is a relative who needs discharging from hospital? Is it the state’s responsibility or is it the responsibility of relatives, if they have any?”... I think the big problem is, there is a national crisis developing about where, about financial ability…there’s no Council nursing homes and care homes and there’s a huge demand for care, that’s your problem. If that’s the problem, how do you solve the problem” (CO_009_family)  “I think it strikes fear in my heart actually to think that they're going to go down this route and think that that is actually going to be successful in most cases. I don't think it would have been successful in my dad's case…the thing that made my dad stay out of hospital was the fact that he had everything he could possibly need. That's his best chance of staying at home and that is how it's turned out, and if those things hadn't been in place for him, he would have ended up back in I'm quite sure” (CO_010_family)  “…because it's not just a case of being medically fit you know they have to they have to have a proper assessment where they are, and proper support once they go back and the way I see it, with the you know the services just are not there anymore, because of the funding cuts” (CO_007_family)  Facilitating ‘Discharge to Assess’  “…honesty, transparency is key, er, and I think that the communication should be on the basis that you’re medically unfit to be at home until we believe you can be discharged….this gives the patient the confidence of knowing that they’re not going to be in hospital any longer than they need to be but equally, they’re not gonna be sent out of hospital with a medical requirement that requires hospitalisation. And I think that transparency is critical because, you know, if you’re in a hospital environment and you’re not confident that you’re getting either the right treatment, the duration of the treatment, or indeed, erm, you’re in longer than you need to be…it undermines the whole process, you know” (CO_001_family)  “I think the expectation, yeah, the expectation should be set and…and then, it’s the nursing [staff] to have responsibility to keep people updated on that…if they’re told that their expectation is that you should be in for three days and they’ve been in for four days already, then it predetermines their expectation. If they’ve been told that they’re likely to be in for five days and that they’re told that they’re gonna be discharged after three days, it then prompts the question, well, why am I getting discharged early, and then it can be used as a positive thing…that we’re able to discharge you ahead of what would typically be a five day stay, we’re able, and make it a positive thing rather than giving people the concern…so setting an expectation right at the beginning [INT: yeah], I think, helps people to deal with what they’ve got to deal with….ah, it augments the information that people already have in their head and it gives them some sort of concrete milestones… a lot of it is psychological for sure” (CO_001_family)  “…the key element to the success of that is excellent communication erm, and, and managing expectation. Erm, and I think it, it, it could probably succeed or fail based on that. And that, for me, would be, would be key and almost, you know, it, it’s, I think it’s done in some respects, you know, when, when you go as an outpatient to hospital, you, they normally, they would send you a letter to say okay, if you go for an endoscopy, or something like that, they would say this is the process, this is how long it takes, this is what you should do…So there’s a predetermined expectation and I think that gives people confidence in a situation… anything that can be done in advance of a discharge, I think would make sense” (CO_001_family) |
