# Supplementary material for: Reducing delayed transfer of care in older people: A qualitative study of barriers and facilitators to shorter hospital stays
Source: Health Expect. 2022 Oct 3;25(6):2628–44. doi: 10.1111/hex.13588 (PMC9700150; doi:10.1111/hex.13588)
Supplement: Supplementary file 2 — Supplementary information. [file HEX-25--s003.docx]

# Additional file 3.

Identified areas for improvement and suggested recommendations

| Areas for improvement | Suggested recommendations |
| --- | --- |
| Care organisation and environment | A staff member dedicated to the discharge process was identified as the ‘ideal’. Geriatricians at Leeds Teaching Hospital Trust have recently piloted a **designated single point of contact for discharge**, and there may be lessons from this project for other Trusts (personal communication).  Being **discharged to a ‘hospital at home’** or ‘virtual ward’ was reassuring for family, and older people found it efficient. Bradford Teaching Hospital has a well-established ‘discharge to assess’ process led by a virtual ward and a multi-disciplinary team working across health and social care organisations to shift ongoing care closer to home. This is recognised as an exemplar and is replicated in many hospital trusts.^35^  Family and older people need help to anticipate ongoing care needs before discharge. Provision of **information about local social and community care options early in admission** would allow time for discussion and clarification with hospital staff. Where GP or outpatient follow-up is needed, referral should be in place before discharge from hospital. Longer term, better links with organisations and partners within the recently mandated integrated care systems will be needed to ensure older people’s complex care needs can be met locally.4^9^  Suggestions to reduce long waits for patient transport included a more organised ‘system’ of allocating people to vehicles to take people home when they are discharged. Recommendations for improving non-urgent patient transport include use of **standard protocols and information technology** such as online booking systems and real-time information sharing between hospital departments.3^1^ |
| Personal preparation and knowledge | There was demand from family and older people for more **transparent communication about expected discharge** and setting expectations in advance. Use of an expected discharge date and a whiteboard to convey updated information has been shown to increase patient satisfaction and communication between patients and staff.3^8^ Other research suggests making staff responsible for updating family and the patient is important and the expected discharge date, treatment and care goals should be provided to family daily.^378^ |
| Information and communication | There was an identified need for a **communications coordinator** to facilitate communication between all parties and allow medically qualified staff to concentrate on patient care. During the COVID-19 pandemic hospitals in the UK introduced Family Liaison Officers when hospital visits were restricted. Recent research supports the potential to standardise these roles to facilitate communication between medical ward teams and families3^9^  Providing older people and family members with a list of questions to ask about discharge could help initiate conversations with staff and obtain information to prepare them better. A **two-way communication checklist** has been shown to improve patient-clinician communication and change the behaviour of the clinician.^40^ The Patients at Care Transitions (PACT) project has also produced resources including a booklet for patients and families to empower them to ask questions and guide discussions with staff.^62^  **Strategic use of written information and predischarge family meetings** could help improve communication and shared decision making in geriatric in-patient care.4^1^ Written information for family members could include discharge preparation and care planning and family meetings could review the hospital stay and what to expect in discharge assessments and after discharge.  **Staff training in communication skills** and having conversations with older people and family members could help to address poor communication and variation in care. A recent review found that nurses’ communication skills can be improved with specific training^63^, and training discharge team members in ‘cooperative communication’ could improve care coordination and communication between health and social care teams at discharge. |
