# Supplementary material for: Reducing delayed transfer of care in older people: A qualitative study of barriers and facilitators to shorter hospital stays
Source: Health Expect. 2022 Oct 3;25(6):2628–44. doi: 10.1111/hex.13588 (PMC9700150; doi:10.1111/hex.13588)
Supplement: Supplementary file 3 — Supplementary information. [file HEX-25--s001.docx]

# Additional file 1.

Content analysis of online care stories: moving from codes to categories and themes

| Codes | Categories n/371 (%) | Themes |
| --- | --- | --- |
| Being refused transport requests | Environment, transfers, moves 155/371 (42) | 1. Deficiencies in the organisation of care |
| Bed moves and ward transfers |  |  |
| Time taken at points in care pathway | Organisation of care 77/371 (21) |  |
| Management shortcomings |  |  |
| Staffing levels |  |  |
| Preparation for discharge | Discharge process 148/371 (40) | 2. Disorganised discharge process |
| Swift discharge |  |  |
| Self-discharge |  |  |
| Coordination and organisation of discharge |  |  |
| Premature discharge |  |  |
| Delays in discharge |  |  |
| Communication between staff | Information and communication 238/371 (64) | 3. Communication is adequate but often deficient |
| Communication with older person |  |  |
| Lack of communication with carer/family |  |  |
| Being cared for | Benefits of hospitalisation 225/371 (61) | 4. What it means to be an older person in hospital |
| Feeling safe |  |  |
| Improved condition |  |  |
| Support with independence |  |  |
| Carer relief |  |  |
| Friendly and positive demeanour of staff | Positive staff care 214/371 (58) |  |
| Staff patience and kindness |  |  |
| Being treated with dignity and respect |  |  |
| Lack of staff compassion | Negative staff care 85/371 (23) |  |
| Treated as unable to make decisions |  |  |
| Poor staff care and attitude |  |  |
| Emotional stress | Risks of hospitalisation 208/371 (56) | 5. Older people’s unmet needs in hospital |
| Deterioration of condition |  |  |
| Unaccommodated needs |  |  |
| Inadequate environment |  |  |
| Stress to carers |  |  |
| Feeling like a burden |  |  |
| Impact of COVID-19 on visiting |  |  |
| Social contact | Family and caregiver support 139/371 (37) | 6. Family members take on liaison and advocacy roles |
| Involvement in health care |  |  |
| Waiting for services |  |  |
| Staff involvement |  |  |

Thematic analysis of interview data: moving from codes to sub-themes and themes

| Codes | Sub-themes | Theme |
| --- | --- | --- |
| Being at home is best for recovery  Its right to shorten hospital stays  Shorter stays are understandable | Family appreciates rationale for shorter stays | 1. Older people and families appreciate the rationale for shorter hospital stays |
| Relief for family | Hospital stay as relief |  |
| Getting care that was needed |  |  |
| In a place where they would get better |  |  |
| Older people were in the right place to get better |  |  |
| Extra resources/staff due to Covid |  |  |
| Older people don’t want to use up hospital resources/ be a burden | Not wanting to be a burden |  |
| Older people view ‘hospital is for sick people’/ fear of dying in hospital |  |  |
| Older people want to be home/ get out quickly/ intolerant |  |  |
| Confinement/ feeling locked in | Boredom and lack of social interaction |  |
| Boredom |  |  |
| Lack of stimulation |  |  |
| Lack of social interaction |  |  |
| Everything is done for you/ institutionalised |  |  |
| Anxious about being a patient |  |  |
| Fear of dying in hospital meant desire to self-discharge |  |  |
| Moved around a lot/ ward to ward | Noise and ward environment not conducive to recovery |  |
| No peace/ a lot of coming and going |  |  |
| Noisy/ mechanical noise/ talking |  |  |
| Lack of sleep |  |  |
| Don’t know who is who/ different cadres of staff |  |  |
| Worried about possessions/ personal items went missing |  |  |
| Comms is lacking between staff | Information amount and quality provided by staff varied | 2. Communication systems designed to fail |
| Staff to patient comms is lacking |  |  |
| Amount of info provided to family varied by staff member |  |  |
| Family has to make repeated calls/ don’t know when to call |  |  |
| Staff don’t always know the patient |  |  |
| Comms is two-way | Important two-way communication |  |
| Family want opportunity to contribute information about their older relative |  |  |
| Family want re-assurance their relative is being looked after | Poor communication undermines confidence in care |  |
| Older people want re-assurance but generally don’t ask for it |  |  |
| Poor communication with staff undermined confidence in care |  |  |
| Poor attitude towards older people undermined confidence in care |  |  |
| Covid restrictions removed ability of doctors to have a ‘quick chat’ with family | COVID-19 exacerbated communication problems |  |
| Some family members felt dismissed by staff | Feeling listened to facilitates care |  |
| Family wanted to help and facilitate care because they know the older person (esp those with dementia) |  |  |
| Comms between hospital and social care settings often lacking | Communication between health and social care lacking |  |
| Basic care was inconsistent in hospital and in community/social services | Care varied depending on staff attitude and personality | 3. Unwanted variation and lack of confidence in care |
| Some staff interested and kind |  |  |
| Other staff were doing it as a job to get paid |  |  |
| Family express lack of confidence in care provided | Family members lacked confidence in care |  |
| Individual staff members often played critical role in discharge process (social worker or discharge nurse) | Individual staff members played critical roles |  |
| Hospital discharge process was experienced in terms of medical needs | Discharge experienced as medical needs assessment | 4. Hospital discharge process caused frustration and anxiety |
| Older people seemed to lack information on the discharge procedure | Older people frustrated by discharge delays |  |
| Discharge process not personal due to Covid | Quicker discharge due to COVID-19 |  |
| Quick and efficient during Covid pandemic |  |  |
| Family members often experienced a relative's hospitalisation as distressing | Family excluded from discharge process |  |
|  | Aspects of care that facilitated discharge |  |
| Didn’t really understand relative's needs or the support available to them | No help to navigate the social care system | 5. Family and older people unprepared for ongoing care needs |
| No help to navigate social care system and entitlements/ especially if self-funded |  |  |
| Disjointed care once back at home or in hands of primary care | Disjointed primary and community care |  |
| Important to assess physical but also mental fitness to go home | Belief that discharge should be medically driven | 6. Factors affecting implementation of ‘discharge to assess’ |
| Older people need to have the confidence that when they are discharged there is provision in place |  |  |
| Set expectations about discharge at the beginning |  |  |
| Care stops in virtual ward | Barriers to implementing discharge to assess |  |
| Where will older people go if they are discharged from hospital and waiting for assessment? |  |  |
| No capacity in community services |  |  |
| Honesty and transparency are key for patient to be confident they’re ready to go home | Facilitating discharge to assess |  |
| Managing expectations is key |  |  |
